# Differential predictive factors for cardiovascular events in patients with or without cancer history

**DOI:** 10.1097/MD.0000000000017602

**Published:** 2019-11-01

**Authors:** Daisuke Sueta, Noriaki Tabata, Satoshi Ikeda, Yuichi Saito, Kazuyuki Ozaki, Kenji Sakata, Takeshi Matsumura, Mutsuko Yamamoto-Ibusuki, Yoji Murakami, Takayuki Jodai, Satoshi Fukushima, Naoya Yoshida, Tomomi Kamba, Eiichi Araki, Hirotaka Iwase, Kazuhiko Fujii, Hironobu Ihn, Yoshio Kobayashi, Tohru Minamino, Masakazu Yamagishi, Koji Maemura, Hideo Baba, Kunihiko Matsui, Kenichi Tsujita

**Affiliations:** a Department of Cardiovascular Medicine, Graduate School of Medical Sciences; b Center for Metabolic Regulation of Healthy Aging, Kumamoto University, Kumamoto; c Department of Cardiovascular Medicine, Nagasaki University Graduate School of Biomedical Sciences, Nagasaki; d Department of Cardiovascular Medicine, Chiba University Graduate School of Medicine, Chiba; e Department of Cardiovascular Biology and Medicine, Niigata University Graduate School of Medical and Dental Sciences, Niigata; f Department of Cardiovascular and Internal Medicine, Kanazawa University Graduate School of Medicine, Kanazawa; g Department of Metabolic Medicine, Faculty of Life Sciences; h Department of Breast and Endocrine Surgery, Graduate School of Medical Sciences; i Department of Urology, Faculty of Life Sciences; j Department of Respiratory Medicine, Graduate School of Medical Sciences; k Department of Dermatology and Plastic Surgery, Faculty of Life Sciences; l Department of Gastroenterological Surgery, Graduate School of Medical Sciences; m Division of Translational Research and Advanced Treatment Against Gastrointestinal Cancer; n Community, Family, and General Medicine, Graduate School of Medical Sciences, Kumamoto University, Kumamoto, Japan.

**Keywords:** atherosclerotic disease, cardiovascular events, malignant disease, obesity paradox, prognostic factors

## Abstract

Although attention has been paid to the relationship between malignant diseases and cardiovascular diseases, few data have been reported. Moreover, there have also been few reports in which the preventive factors were examined in patients with or without malignant disease histories requiring percutaneous coronary intervention (PCI).

This was a retrospective, single-center, observational study. A total of 1003 post-PCI patients were divided into a malignant group, with current or past malignant disease, and a nonmalignant group. The primary endpoint was a composite of cardiovascular death, nonfatal myocardial infarction, stroke, revascularization, and admission due to heart failure within 5 years of PCI. Kaplan–Meier analysis showed a significantly higher probability of the primary endpoint in the malignant group (*P* = .002). Multivariable Cox hazard analyses showed that in patients without a history of malignant, body mass index (BMI) and the presence of dyslipidemia were independent and significant negative predictors of the primary endpoint (BMI: hazard ratio [HR] 0.73, 95% confidence interval [CI] 0.53–0.99, *P* = .041; prevalence of dyslipidemia: HR 0.72, 95% CI 0.52–0.99, *P* = .048), and the presence of multi-vessel disease (MVD) and the prevalence of peripheral artery disease (PAD) were independent and significant positive predictors of the primary endpoint (prevalence of MVD: HR 1.68, 95% CI 1.18–2.40, *P* = .004; prevalence of PAD: HR 1.51, 95% CI 1.03–2.21, *P* = .034). In patients with histories of malignancy, no significant independent predictive factors were identified.

Patients undergoing PCI with malignancy had significantly higher rates of adverse cardiovascular events but might not have the conventional prognostic factors.

## Abstract 2

Supplemental Digital Content is available in the text

## 1 Introduction

The original concept of onco-cardiology was developed on the basis of cardiotoxicity associated with anticancer treatment^[1–3]^; however, recently, the number of cases in which atherosclerotic cardiovascular diseases coexist with malignant diseases has increased, and increasing attention is being paid to the relationship between malignant diseases and cardiovascular diseases, based on the long-term surveillance of cancer survivors.^[4–6]^ Several studies have described a high risk of cardiovascular disease events in cancer survivors.^[7–15]^ Similarly, a history of cardiovascular disease also confers a higher risk of cancer.^[16–19]^ Thus, this relationship has been receiving attention as a new aspect of onco-cardiology,^[20–22]^ and these concepts were comprehensively reviewed recently.^[23,24]^ We have observed that many malignant diseases and atherosclerotic diseases coexist in university hospitals (Supplemental Material) and have proposed that university hospitals may represent a microcosm of the future population.^[12]^ However, to the best of our knowledge the risk of cardiac events in coronary artery disease (CAD) patients with malignancy has not been well elucidated.

Moreover, our previous study^[25]^ on the cardiovascular events in patients with comorbid malignancies and CADs was limited by the 1-year follow-up period, and long-term follow-up is required. In the present study, we; therefore, examined the long-term clinical outcomes of cancer patients undergoing percutaneous coronary intervention (PCI).

Accumulating longitudinal studies have suggested that obesity is an independent predictor of CAD,^[26,27]^ whereas it was also reported that body mass index (BMI) was inversely correlated with mortality in CAD patients.^[28–31]^ This led to the proposal of the concept called the “obesity paradox,” and there not yet any definite conclusions.^[32]^ We also examined whether various factors, including the “obesity paradox,” contributed to the presence or absence of malignant diseases.

## 2 Methods

This study was a retrospective, single-center, observational study. The study was registered at the University Hospital Medical Information Network Clinical Trial Registry (UMIN000028652). This research was a collaborative study by the Consortium of Six National Universities in Japan (Nagasaki University, Chiba University, Kanazawa University, Niigata University, Okayama University, and Kumamoto University).

### 2.1 Ethics statement

All procedures were conducted in accordance with the Declaration of Helsinki and its amendments. The study protocol was approved by the institutional review boards of each institution.

### 2.2 Definition of malignant diseases

A detailed description is available in the Supplemental Material

### 2.3 Definition of atherosclerotic diseases

Atherosclerotic diseases were defined as any clinical evidence of diseases thought to be due to atherosclerosis (ie, ischemic heart disease, ischemic heart failure, peripheral artery disease [PAD], aortic valve stenosis [excluding congenital bicuspid valve], arteriosclerotic aneurysm, arterial dissection, noncardiac cerebral infarction, and nephrosclerosis).

### 2.4 Study design

**Figure 1:**
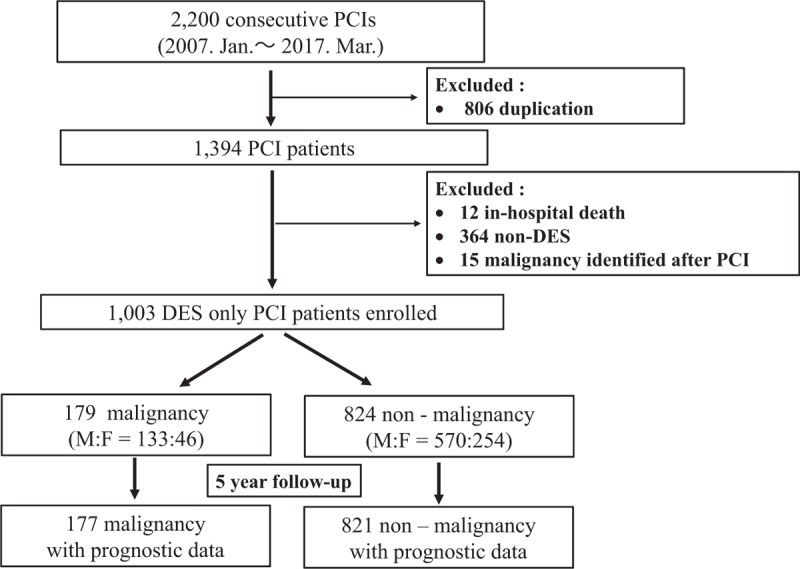
Flow chart of the present study. DES = drug-eluting stent, F = female, M = male, PCI = percutaneous coronary intervention.

We reviewed the medical records and defined patients with malignancies as those with medical histories of previous and current malignant diseases. The present study is a sub-analysis of our previous study.^[25]^ This study included 2200 consecutive PCI patients treated at the Kumamoto University Hospital between January 2007 and March 2017. We excluded the following patients: 806 duplicate patients; 12 patients who succumbed to in-hospital death; 364 patients with bare-metal stents (BMSs), balloon angioplasty, aspiration, PCI failure, and excimer laser coronary angioplasty? and 15 patients who were identified as having a malignancy after PCI. The remaining 1003 drug-eluting stent (DES)-only PCI patients were enrolled (Fig. 1). Acute coronary occlusion, which was a problem in the balloon catheter-only era, has been overcome by the advent of the BMS. With the development of the DES, the incidence of in-stent restenosis, which was a problem associated with BMS use, was reduced by several percentage points.^[25]^ Recently, we do not have many opportunities to deploy BMS, and we use DES even for patients with acute coronary syndrome. Moreover, the latest guidelines on myocardial revascularization recommend that DES be used instead of BMS for any PCI.^[33]^ Hence, it was necessary to establish a unified study of DES. Therefore, in this study, we decided to examine the effect of composite cardiovascular events, including restenosis on malignant diseases in DES-only cases. We divided the enrolled patients into 2 groups according to the presence of malignant diseases: the malignancy group and the nonmalignancy group (Fig. 1). The detailed definition of malignancy is described above.

### 2.5 Clinical parameters

The clinical parameters were described previously.^[12,25,34]^ In brief, baseline demographic data, cardiovascular risk factors, and medications on discharge after PCI were documented. Hypertension was defined as blood pressure >140/90 mm Hg or taking antihypertensive medication. We defined diabetes mellitus (DM) as the presence of symptoms of diabetes and a casual plasma glucose concentration ≥200 mg/dL, a fasting plasma glucose concentration ≥126 mg/dL, and a 2-hours plasma glucose concentration ≥200 mg/dL on a 75-g oral glucose tolerance test, or taking medication for DM. Dyslipidemia was defined as low-density lipoprotein ≥140 mg/dL (≥3.63 mmol/L), high-density lipoprotein <40 mg/dL (1.04 mmol/L), or triglycerides ≥150 mg/dL (≥1.7 mmol/L). Chronic kidney disease (CKD) was defined as an estimated glomerular filtration rate (eGFR) <60 mL/min/1.73 m^2^.^[35]^ Current smoking status was determined via an interview. Acute coronary syndrome was defined as either an acute myocardial infarction (MI) (ST-segment elevation MI or non-ST-segment elevation MI) or unstable angina pectoris. We used the latest universal definition of MI in this study.^[36]^ Patients with past or current intermittent claudication associated with an ankle-brachial index value of <0.9 in either leg were categorized as having PAD. Patients with previous ischemic stroke or transient ischemic attack were defined as having previous stroke. We recorded coronary lesions as the number of diseased coronary vessels. We defined the stenosis of more than 75% per the report from the American Heart Association^[37]^ to be significant and indicative of the need for primary PCI.

### 2.6 Follow-up and clinical events

The observations were performed by investigators who were blind to the patients allocation in this study. The agreement regarding the assessment of outcomes was achieved with the attainment of consensus among multiple evaluators in cases where doubts arose regarding endpoint decisions.

After PCI, patients were followed-up prospectively at outpatient clinics for 5 years or until an endpoint occurred. The primary endpoint was a composite of cardiovascular death, nonfatal MI, stroke, revascularization (target lesion revascularization [TLR] or non-TLR-revascularization) and admission due to heart failure up to 5 years (the median follow-up period was 343 days). At the 5-year follow-up visit, we measured the total number of endpoint events and stopped the analysis. Cardiovascular events were ascertained by reviewing the medical records and were confirmed by direct contact with the patients, their families, or their physicians. Cardiovascular death was defined as death due to MI or congestive heart failure or as documented sudden cardiac death. We defined revascularization (TLR or non-TLR- revascularization) as clinically-driven revascularization; specifically, revascularization was confirmed when the follow-up coronary angiography revealed restenosis or lesion progression and findings such as the presence of chest pain or positive results for stress myocardial scintigraphy or fractional flow reserve. There were no differences in the enforcement rate of follow-up coronary angiography between the 2 groups. For patients who suffered more than 1 cardiovascular event, only the first event was counted.

### 2.7 Sample size calculation

Our previous study^[12]^ showed approximately 20% of patients had malignant diseases in our institute. Accordingly, we planned a study of independent cases and controls with 5 controls per case. Our pilot data^[12]^ indicated that the probability of exposure among controls was 0.2. If the true probability of exposure among cases was 0.3, we would need to study 169 case-patients and 845 control patients to be able to reject the null hypothesis that the exposure rates for cases and controls are equal with a probability (power) of 0.8. The type I error probability associated with this test of the null hypothesis is 0.05.

### 2.8 Statistical analysis

The Shapiro–Wilk test was used to assess the normal distribution of continuous data. Continuous variables with normal distributions are expressed as the means ± standard deviation, whereas those with skewed distributions are expressed as the median values with their interquartile ranges. Categorical data are presented as numbers or percentages. Differences between 2 groups were tested using a Fisher exact test or a Chi-squared test for categorical variables, as appropriate. Differences in continuous variables were analyzed with analysis of variance or the Mann–Whitney *U* test. We used the Kaplan–Meier method to estimate the cardiovascular event probabilities at 1825 days and a log-rank test to compare the distributions of survival times among the groups. Cox proportional hazard models were used to calculate hazard ratios (HRs). Multivariable analyses were performed using forced inclusion methods, and predictors of clinical outcomes that were identified through univariable analyses were tested in a multivariable analysis (*P* < .05). A *P*-value < .05 was considered to denote statistical significance. Statistical analyses were performed using SPSS version 25 (IBM Inc, Armonk, NY).

## 3 Results

### 3.1 Study population, the prevalence of comorbidities among the study participants and malignant disease incidence

Among 1003 enrolled DES-only patients, 17.8% (n = 179) of the patients (18.9% [n = 133] of males and 15.3% [n = 46] of females) had a past medical history of malignant disease. Data for 998 PCI patients were available for the analysis of subsequent adverse cardiovascular events (data for 5 patients were unavailable). The clinical characteristics of the study participants were described previously.^[25]^ In brief, compared with patients without malignancy, patients with malignancy were older (patients with malignancy averaged 73.09 years old, and patients without malignancy averaged 69.75 years old) and had lower prevalence rates of dyslipidemia, current tobacco use, and previous stroke. Concerning the coronary and PCI details, we found no significant differences in single, double, triple, and left main trunk lesions between the malignant and nonmalignant groups. In addition, there were no significant differences in the frequency of medication usage (statins; angiotensin-converting enzyme inhibitors or angiotensin receptor blockers; beta-blockers; and proton pump inhibitors) upon discharge between the groups. In both groups, aspirin was used in approximately 97% of the patients, and P2Y_12_ inhibitors were used in approximately 94%; statistically there were no significant differences between the 2 groups. These results rejected the possibility of reduced use of dual antiplatelet therapy in patients with malignancy. The clinical characteristics stratified by the presence of the event in the study participants are shown in Table 1. There were no significant differences among the patient characteristics between the event-positive (n = 54) and event-negative groups (n = 123) for patients with malignant diseases. For patients without malignant diseases, patients who experienced an event had lower BMI values, abdominal circumferences, eGFRs and ratios of single vessel diseases. Patients who experienced an event had higher prevalences of Diabetes and CKD and previous PAD.

**Table 1:**
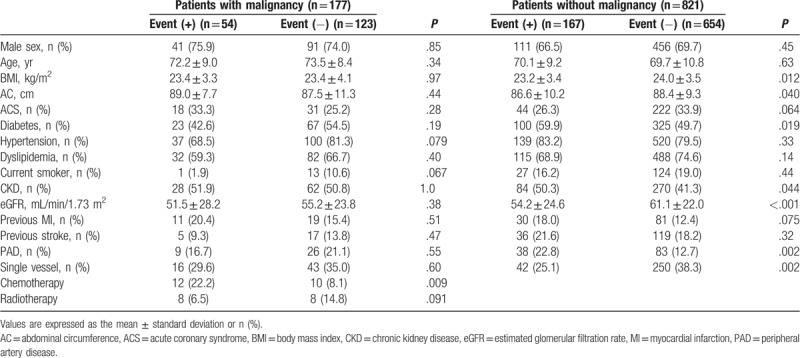
Clinical parameters of the study participants at baseline stratified by malignancy status.

The types of malignancies were described previously.^[25]^ In brief, the top 4 most common malignancies were prostate, colorectum, liver, and lung cancers.

### 3.2 Primary endpoints at the follow-up

**Figure 2:**
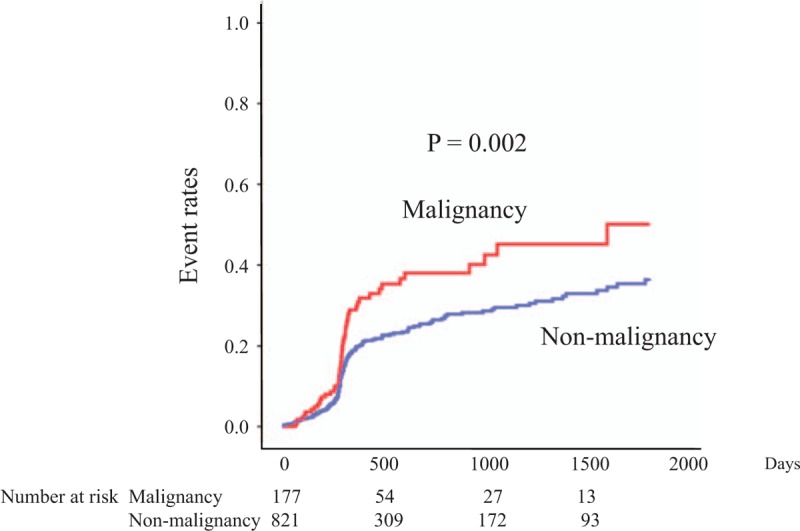
Kaplan–Meier curves for the primary endpoint. Kaplan–Meier analysis demonstrated a significantly higher probability of adverse outcomes in patients with malignancies (malignancy group) than in patients without malignancies (nonmalignancy group) (*P* = .002).

During the follow-up period (median, 343 days), 221 (22.1%) of the patients experienced an adverse cardiovascular event (30.5% of the patients in the malignancy group and 20.3% of the patients in the nonmalignancy group). Kaplan–Meier analysis demonstrated a significantly higher probability of adverse outcomes in patients with malignancies than in the patients without malignancies (*P* = .002; Fig. 2). Details of the cardiovascular events are shown in Table 2, which shows that we found significantly higher rates of cardiovascular death and revascularization in the patients with malignancies than in the patients without malignancies (*P* = .003 and *P* = .02, respectively).

**Table 2:**
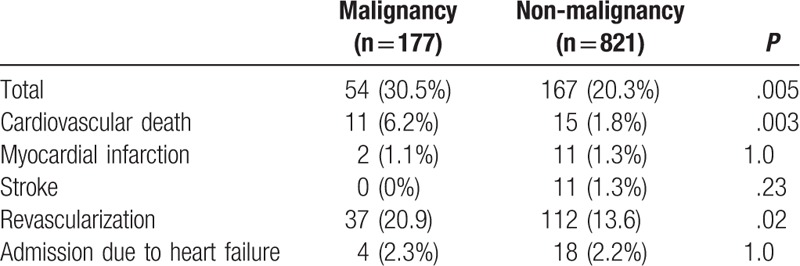
Cardiovascular events according to malignant disease history.

### 3.3 Cox proportional hazards analyses for the primary endpoint

We carried out univariable and multivariable Cox proportional hazards analyses for the primary endpoints (Table 3). Multivariable Cox proportional hazard analysis was conducted with the forced inclusion model including conventional risk factors and showed that malignancy was an independent predictor of the primary endpoint (HR, 1.49; 95% confidence interval [CI], 1.10–2.04; *P* = .011) and that BMI (above median = 23.52 kg/m^2^) and the prevalence of dyslipidemia were independent and significant negative predictors of the primary endpoint (BMI: HR 0.74, 95% CI 0.56–0.96, *P* = .025; prevalence of dyslipidemia: HR 0.75, 95% CI 0.56–1.00, *P* = .048)

**Table 3:**
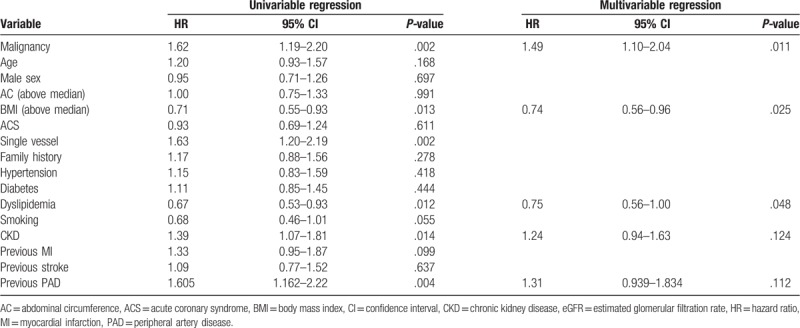
Cox proportional hazards regression analyses for clinical outcome within 5-year follow-up.

We next performed univariable and multivariable Cox proportional hazards analyses for the primary endpoint in the malignancy and nonmalignancy groups (Table 4). In patients without histories of malignancy, BMI (above median = 23.52 kg/m^2^) and the prevalence of dyslipidemia were independent and significant negative predictors of the primary endpoint (BMI: HR 0.73, 95% CI 0.53–0.99, *P* = .041; prevalence of dyslipidemia: HR 0.72, 95% CI 0.52–0.99, *P* = .048), while the prevalence of multi-vessel disease (MVD) and the prevalence of PAD were independent and significant positive predictors of the primary endpoint (prevalence of MVD: HR 1.68, 95% CI 1.18–2.40, *P* = .004; prevalence of PAD: HR 1.51, 95% CI 1.03–2.21, *P* = .034). In patients with histories of malignancy, no significant independent factors were identified.

**Table 4:**
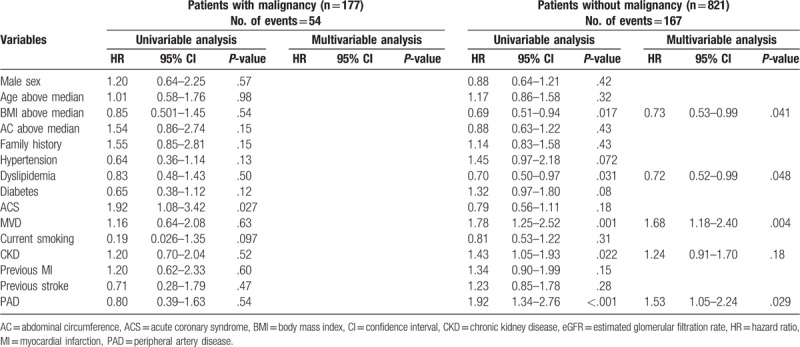
Predictors of clinical outcomes using the Cox proportional hazard model for the malignancy and non-malignancy groups.

### 3.4 Effect of cancer treatment on cardiovascular events

It has been suggested that chemotherapy and radiotherapy used in cancer treatments sometimes induce cardiotoxicity and cause heart failure, which was the original concept behind onco-cardiology/cardio-oncology. In the present study, 22 and 16 of 177 cancer patients had histories of chemotherapy and radiotherapy, respectively. Of the 177 patients with prognostic data, there was a significant difference in the occurrence of cardiovascular events between the chemotherapy experienced and naïve groups (chemotherapy experienced: 12/22 [54.5%]; chemotherapy naïve: 42/155 [27.1%], *P* = .009), while there was no significant difference in the occurrence of cardiovascular events between the radiotherapy experienced and naïve groups (radiotherapy experienced: 8/54 [14.8%]; radiotherapy naïve: 8/123 [6.5%], *P* = .091).

## 4 Discussion

“Onco-cardiology” or “cardio-oncology” has been used in reference to cardiotoxicity during the treatment of malignant diseases.^[1,2]^ While the adverse effects associated with recent progress in chemotherapeutic cancer treatments are concerning,^[38,39]^ the relationship between malignant diseases and cardiovascular diseases has also attracted attention.^[6,21,22]^ However, the comorbidity of malignant diseases and cardiovascular diseases has never been investigated extensively; therefore, we conducted this multicenter collaborative surveillance. As shown in Supplemental Figure 1, the statistically significant coexistence of malignant diseases and atherosclerotic diseases was observed only in clinical departments that deal with atherosclerotic diseases at university hospitals (Group C).

The prevalence of obesity has dramatically increased not only in developed countries but also in developing countries,^[40]^ and it has become a social problem worldwide. Although the relationship between obesity and CAD has been deemed to be due to cardiovascular risk factors such as hypertension and DM related to obesity, it has been previously reported that obesity itself was an independent predictor of CAD in longitudinal cohort studies.^[26,27]^ However, in 2002, Gruberg et al reported that BMI was inversely correlated with mortality in CAD patients; this phenomenon was different from general perception, and therefore it led to the proposal of the concept called the “obesity paradox.”^[28]^ After that, similar reports^[29–31]^ were forthcoming. It was thought that the residual confounding factors contribute to this relationship,^[41,42]^ but the mechanism has not been elucidated. As shown in Table 2, it was suggested that the presence of malignant disease might contribute to the discrepancy, which was possibly one of residual confounding factor. Moreover, the occurrence rate of events varies depending on the presence or absence of malignancy as previously reported.^[12]^ Conventional prognostic factors, such as renal function, MI history, and PAD history, have not been applied to the malignant disease group as BMI and abdominal circumference have. The reason conventional prognostic factors do not apply in the malignant disease group is not clear, but the following reasons can be suggested. Because malignant diseases and atherosclerotic diseases share certain risk factors,^[43–46]^ it would be reasonable to expect malignant disease patients to have atherosclerotic diseases. Furthermore, malignant diseases^[47–49]^ and atherosclerosis lesions^[50–52]^ are both characterized by inflammation. We have already reported that the combination of malignancy and high high-sensitivity C-reactive protein levels has been associated with significantly higher incidences of cardiovascular events.^[12]^ Thus, we speculate that local malignancies increase vascular wall inflammation by increasing the levels of various inflammatory cytokines^[53–55]^ and that this circulatory inflammation causes progressive arteriosclerosis. As shown in the present study, conventional prognostic factors such as renal dysfunction and PAD history do not apply to the malignant disease group as BMI and abdominal circumference do (Table 1). Hence, 1 possibility why conventional prognostic factors do not apply to the malignant disease group is that malignancy itself might be a residual risk factor for cardiovascular events. Recently, the concept of clonal hematopoiesis of indeterminate potential^[56]^ was proposed for myeloid malignancies, and Libby and Ebert comprehensively reviewed its contribution to cardiovascular risks.^[57]^ Hence, we believe that the important mechanisms underlying this association are common risk factors, inflammation, and clonal factors.

The results obtained in the present study (Fig. 2) were primarily caused by revascularization (Table 2). In Japan, it is common to perform follow-up coronary angiographies 8 to 12 months after PCI,^[58]^ and the results of these procedures were considered in the present study. We have already reported the details of this mechanism.^[12]^ It is well known that radiotherapy, especially thoracic radiotherapy, promotes atherosclerosis.^[59]^ In this study, the possibility of a synergistic relationship between radiotherapy and revascularization was proposed.

## 5 Study limitations

The present study has several limitations. First, this study was a retrospective single-center observational study. Despite the relatively small number of patients involved, we included patients from a large catchment area and, thus, included a high number and a wide range of cancers among the patients studied, reflecting the broader incidences observed nationally and/or worldwide. In particular, there is a possibility that predictive factors were not identified in the malignancy group due to the small sample size and low statistical power. Second, the possibility that the clinical endpoints observed in this study were influenced by patient medications, the use of anticancer agents or thoracic irradiation cannot be ignored. Third, it is not clear whether patients with both malignant diseases and atherosclerotic diseases have worse prognoses. Fourth, it is unclear which factors contribute or to what extent specific factors contribute to the development of atherosclerotic diseases and the promotion of malignant diseases. Moreover, accumulating clinical evidence has shown that patients with malignancies, compared with those without malignancies, have a higher likelihood of embolism, such as pulmonary embolism or coronary embolism due to thrombus or tumor tissue/mass, which might partly explain why patients with malignancies have a higher risk of cardiovascular events after PCI. Fifth, we set the endpoint as the time when the first event occurred and many were revascularizations. Revascularization is often resulted from routine follow-up coronary angiography and may not be clinically driven. Therefore, the possibility that many more clinically relevant outcomes are being missed cannot be denied. Finally, we did not set a control group (the history of malignancy only group). Thus, it is difficult to conclude the role of malignancy itself in future cardiovascular events. Therefore, further pathophysiological and molecular physiological studies, including animal experiments, are warranted. Additional detailed, prospective, large-scale, long-term surveillance may be needed to verify our theories.

## 6 Conclusion

Despite the limitations mentioned above, the results of this study demonstrate the following: patients with malignancies have significantly higher rates of adverse cardiovascular events but might not have the conventional prognostic factors, possibly due to the mechanism underlying the “obesity paradox.”

## Acknowledgments

The authors thank all of the study collaborators for their devoted retrospective clinical records observations. The authors also thank all the paramedical staff and clinical secretaries for their kind support during this work.

## Author contributions

SI has received honoraria from Daiichi Sankyo Co, Ltd, and has received grants from Astellas Pharma Inc; Bayer Yakuhin Ltd; Nippon Boehlinger Ingelheim Co, Ltd; Boston Scientific Japan K. K.; Daiichi Sankyo Co, Ltd; Eisai Co, Ltd; MSD K.K.; Bristol-Myers Squibb K.K.; Actelion Pharmaceuticals Japan Ltd; Teijin Pharma Ltd; Japan Lifeline Co, Ltd; Takeda Pharmaceutical Co, Ltd; Medtronic Japan Co Ltd; and Mitsubishi Tanabe Pharma. KO has received grants from Astellas Pharma Inc; Boston Scientific Japan K.K.; Mitsubishi Tanabe Pharma; and MSD K.K. SF has received grants from Ono Pharmaceutical Co, Ltd; Merck Sharp & Dohme Co, Ltd; Bristol-Myers Squibb, Chugai Pharmaceutical Co, Ltd; Novartis Pharma K.K. and Ltd; and Maruho Co, Ltd. NY belongs to departments supported by Chugai Pharmaceutical Co, Ltd and Yakuruto Honsya Co, Ltd. TK has received honoraria from Astellas Pharma Inc and Takeda Pharmaceutical Co, Ltd, and has received grants from Kyowa Hakko Kirin Co, Ltd; Eisai Co, Ltd; Astellas Pharma Inc; Taiho Pharmaceutical Co, Ltd; Takeda Pharmaceutical Co, Ltd; Nippon Kayaku Co, Ltd; Ono Pharmaceutical Co, Ltd; Sanofi K.K.; Pfizer Japan, Inc; Asahi Kasei Pharma Corporation. EA received grants from Astellas Pharma; AstraZeneca, Daiichi Sankyo; Kowa Pharmaceutical; Mitsubishi Tanabe Pharma; Ono Pharmaceutical; Pfizer Japan; Nippon Boehringer Ingelheim; Novartis Pharma; Novo Nordisk Pharma; Sanofi; Taisho Toyama Pharmaceutical; and Takeda Pharmaceutical and personal fees from Astellas Pharma; AstraZeneca; Daiichi Sankyo; Eli Lilly; Kowa Pharmaceutical; Mitsubishi Tanabe Pharma; MSD; Nippon Boehringer Ingelheim; Novartis Pharma; Novo Nordisk Pharma; Ono Pharmaceutical; Sanofi; Taisho Toyama Pharmaceutical; and Takeda Pharmaceutical. HIW has received honoraria from AstraZeneca K.K.; Chugai-Roche Co, Ltd; and Takeda Pharmaceutical Co, Ltd, and has received grants from AstraZeneca K.K.; Chugai-Roche Co, Ltd; Daiichi Sankyo Co, Ltd; Eisai Co, Ltd; Kowa Pharmaceutical Co, Ltd, and Pfizer Japan, Inc. YK has received honoraria from Amgen Astellas BioPharma K.K.; AstraZeneca; Bristol-Myers Squibb; Daiichi Sankyo Co, Ltd; Boehringer Ingelheim Japan; and Sanofi K.K. and has received grants from Abbott Vascular Japan; Astellas Pharma Inc; Boehringer Ingelheim Japan; Boston Scientific Japan K.K.; Daiichi Sankyo Co, Ltd; Japan Lifeline; Medtronic; Nipro; Otsuka Pharmaceutical; Pfizer Japan, Inc; Sanofi K.K.; Sumitomo Dainippon Pharma; Takeda Pharmaceutical Co, Ltd; and Terumo. TM discloses lecture fees from Amgen Astellas BioPharma K.K.; Sanofi K.K.; Nippon Boehringer Ingelheim, Co, Ltd; Mitsubishi Tanabe Pharma Corporation; MSD; Bayer Yakuhin Ltd; Daiichi Sankyo Co, Ltd; and Takeda Pharmaceutical Co, Ltd, as well as research funds form Boehringer Ingelheim, Co, Ltd; Mitsubishi Tanabe Pharma Corporation; Astellas Pharma Inc; Daiichi Sankyo Co, Ltd; Pfizer Japan Inc; Bayer Yakuhin Ltd; Takeda Pharmaceutical Co, Ltd; Bristol-Myers Squibb; Novartis Pharma K.K.; and AstraZeneca K.K. MY has received honoraria from Daiichi Sankyo Co, Ltd and Sanofi K.K. KM has received honoraria from Daiichi Sankyo Co, Ltd; MSD K.K.; Nippon Boehlinger Ingelheim Co, Ltd; and Actelion Pharmaceuticals Japan Ltd and has grants from Astellas Pharma Inc; Bayer Yakuhin Ltd; Nippon Boehlinger Ingelheim Co, Ltd; Boston Scientific Japan K.K.; Daiichi Sankyo Co, Ltd; Eisai Co, Ltd; MSD K.K.; Bristol-Myers Squibb K.K.; Actelion Pharmaceuticals Japan Ltd; Teijin Pharma Ltd; Japan Lifeline Co, Ltd; Takeda Pharmaceutical Co, Ltd; Medtronic Japan Co, Ltd; and Mitsubishi Tanabe Pharma. HB has received honoraria from Eli Lilly Japan K.K. and Ono Pharmaceutical Co, Ltd and has received grants from Chugai Pharmaceutical Co, Ltd; Covidien Japan Inc; Eli Lilly Japan K.K.; Shionogi & Co, Ltd; Toyama Chemical Co, Ltd; Taiho Pharmaceutical Co, Ltd; Yakult Honsha Co, Ltd; Takeda Pharmaceutical Co, Ltd; Shin Nippon Biomedical Laboratories, Ltd; Merck Serono Co, Ltd; Novartis-Pharma K.K.; and Johnson & Johnson K.K. KT has received honoraria from Amgen Astellas BioPharma K.K.; Bayer Yakuhin, Ltd; Daiichi Sankyo Co, Ltd; MSD K.K.; and Sanofi K.K. and has received grants from AstraZeneca K.K.; Astellas Pharma Inc; Bayer Yakuhin, Ltd; Boehringer Ingelheim Japan; Boston Scientific Japan K.K.; Chugai Pharmaceutical Co, Ltd; Daiichi Sankyo Co, Ltd; Eisai Co, Ltd; Kowa Pharmaceutical Co, Ltd; Mitsubishi Tanabe Pharma; MSD K.K.; Pfizer Japan Inc; Sanofi K.K.; Shionogi & Co, Ltd; and Takeda Pharmaceutical Co, Ltd. The remaining authors have nothing to disclose.

**Conceptualization:** Daisuke Sueta, Noriaki Tabata.

**Data curation:** Daisuke Sueta, Noriaki Tabata, Satoshi Ikeda, Yuichi Saito, Kazuyuki Ozaki, Kenji Sakata, Takeshi Matsumura, Mutsuko Yamamoto-Ibusuki, Yoji Murakami, Takayuki Jodai, Satoshi Fukushima, Naoya Yoshida.

**Formal analysis:** Daisuke Sueta, Noriaki Tabata.

**Investigation:** Daisuke Sueta, Noriaki Tabata, Tomomi Kamba, Eiichi Araki, Hirotaka Iwase, Kazuhiko Fujii, Hironobu Ihn, Yoshio Kobayashi, Tohru Minamino, Masakazu Yamagishi, Koji Maemura, Hideo Baba.

**Methodology:** Daisuke Sueta.

**Project administration:** Daisuke Sueta, Kenichi Tsujita.

**Resources:** Daisuke Sueta.

**Software:** Daisuke Sueta, Noriaki Tabata.

**Supervision:** Hideo Baba, Kenichi Tsujita.

**Validation:** Noriaki Tabata, Kunihiko Matsui, Kenichi Tsujita.

**Visualization:** Daisuke Sueta, Noriaki Tabata, Kunihiko Matsui.

**Writing – original draft:** Daisuke Sueta.

**Writing – review and editing:** Kenichi Tsujita.

## Supplementary Material

Supplemental Digital Content
