# Supplementary material for: Differential predictive factors for cardiovascular events in patients with or without cancer history
Source: Medicine (Baltimore). 2019 Nov 1;98(44):e17602. doi: 10.1097/MD.0000000000017602 (PMC6946347; doi:10.1097/MD.0000000000017602)

**Appendix**

**Study Collaborators:**

Eiichiro Yamamoto^1^, Seiji Takashio^1^, Yuichiro Arima^1^, Satoshi Araki^1^, Kenshi Yamanaga^1^, Masanobu Ishii^1^, Kenji Sakamoto^1^, Hisanori Kanazawa^1^, Koichiro Fujisue^1^, Shinsuke Hanatani^1^, Hirofumi Soejima^1^, Seiji Hokimoto^1^, Yasuhiro Izumiya^1^, Sunao Kojima^1^, Hiroshige Yamabe^1^, Koichi Kaikita^1^, Tomoko Tanaka^1^, Megumi Yamamuro^1^, Koichi Sugamura^1^, Naohiro Komura^1^, Takashi Miyazaki^1^, Tomonori Akasaka^1^, Yoshiro Onoue^1^, Hisao Ogawa^1^, Katsuo Noda^2^, Tomohiro Sakamoto^3^, Kazuteru Fujimoto^4^, Yoshihiro Yamada^5^, Toyoki Hirose^6^, Naritsugu Sakaino^7^, Takashi Komorita^8^, Mitsutoshi Miura^9^, Hiroaki Kusaka^10^, Shinji Tayama^11^, Shinichi Nakamura^12^, Ikuo Misumi^13^, Takanori Tokitsu^14^ and Yoshihiro Hirata^15^.

1. Department of Cardiovascular Medicine, Graduate School of Medical Sciences, Kumamoto University, Kumamoto

2. Division of Cardiology, Kumamoto Central Hospital, Kumamoto

3.Cardiovascular Center, Kumamoto Saiseikai Hospital, Kumamoto

4.Division of Cardiology, National Hospital Organization Kumamoto Medical Center, Kumamoto

5. Division of Cardiology, Fukuoka Tokushukai Hospital, Fukuoka

6.Division of Cardiology, Minamata City Hospital and

Medical Center, Minamata

7. Division of Cardiology, Amakusa Regional Medical Center, Amakusa

8. Division of Cardiology, Kumamoto Rosai Hospital, Yatsushiro

9. Division of Cardiology, Shinbeppu Hospital, Beppu

10. Miyazaki Prefectural Nobeoka Hospital, Nobeoka

11. Division of Cardiology, Kumamoto General Hospital, Yatsushiro

12. Division of Cardiology, Hitoyoshi General Hospital, Hitoyoshi

13. Division of Cardiology, Saishunsou Kumamoto Hospital, Kikuchi

14. Division of Cardiology, Tamana Central Hospital, Tamana

15. Division of Cardiology, Kumamoto Regional Medical Center, Kumamoto

**Supplementary Material for Online Publication**

***The definition of malignant diseases***

The definition of malignancy for this study is a past history or present diagnosis of the following diseases:

1) Cancer originating from any hematopoietic organ. Representative cancers include leukemia, malignant lymphoma, and myeloma.

2) Cancer originating from epithelial cells (epithelial tumor). Representative cancers (carcinomas) generated from epithelial cells include lung cancer, breast cancer, stomach cancer, colorectal cancer, uterine cancer, ovarian cancer, and head and neck cancer (laryngeal cancer, pharyngeal cancer, and tongue cancer).

3) Sarcoma originating from nonepithelial cells. Representative sarcomas include osteosarcoma, chondrosarcoma, rhabdomyosarcoma, leiomyosarcoma, fibrosarcoma, liposarcoma and angiosarcoma.

***Multicenter Comorbidity Surveillance***

This study included 5,798 consecutive patients, admitted between October 2016 and December 2016 in the departments described below. We excluded 315 duplicate patients, and the remaining 5,483 patients were enrolled (Supplemental Figure S1). These patients were admitted across 25 departments (11 departments across 5 universities and 14 departments across 14 community hospitals). The 14 departments across the 14 community hospitals were all cardiovascular divisions with capacities of more than one hundred beds around Kumamoto Prefecture. Detailed information regarding the 14 community hospitals can be found in the Appendix. Patients from community hospitals were defined as belonging to Group A (Supplemental Figure 1). The remaining 11 departments came from 5 university hospitals (Nagasaki University, Chiba University, Kanazawa University, Niigata University and Kumamoto University), with 1,303 patients admitted to 6 departments that primarily handle atherosclerotic diseases (the Departments of Cardiovascular Medicine and Metabolic Medicine). A further 927 patients were admitted to 5 departments that primarily handle malignant diseases (the Departments of Breast and Endocrine Surgery, Urology, Respiratory Medicine, Dermatology and Gastroenterological Surgery at Kumamoto University Hospital). Patients admitted to hospitals that primarily treat malignant diseases were defined as belonging to Group B (Supplemental Figure 1). We reviewed the medical records and defined malignancy patients as those with medical histories of previous and current malignant diseases. Patients admitted to hospitals that primarily treat atherosclerotic diseases were defined as belonging to Group C (Supplemental Figure 1).

***Study population and the prevalence of comorbidities among the study participants***

In Group A, the prevalence of malignant diseases in atherosclerotic diseases and in non-atherosclerotic diseases were 12.4% and 12.5%, respectively (p=1.00). In Group B, the prevalence of atherosclerotic diseases in malignant diseases and in non-malignant diseases were 11.1% and 12.9%, respectively (p=0.45). In Group C, the prevalence of malignant diseases in atherosclerotic diseases and in non-atherosclerotic diseases were 23.0% and 12.8%, respectively (p<0.01).

**Supplemental Figure 1. Flow chart of Multicenter Comorbidity Surveillance**


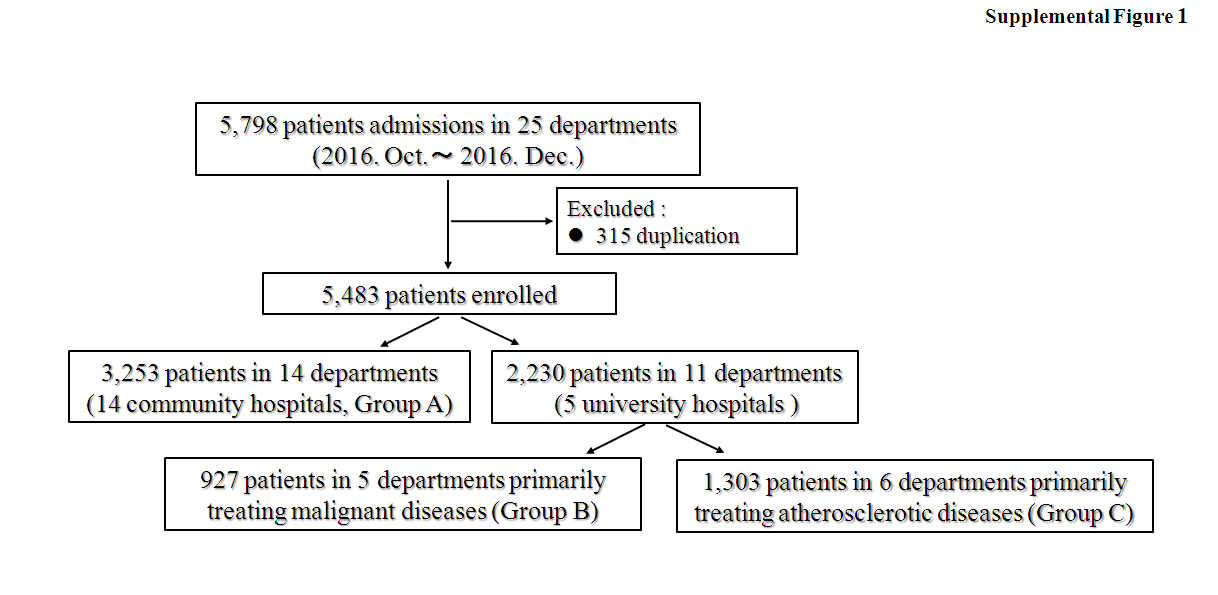

Supplement: Supplemental Digital Content [file medi-98-e17602-s001.docx]
